# Acquired *RAS* or *EGFR* mutations and duration of response to EGFR blockade in colorectal cancer

**DOI:** 10.1038/ncomms13665

**Published:** 2016-12-08

**Authors:** Beth O. Van Emburgh, Sabrina Arena, Giulia Siravegna, Luca Lazzari, Giovanni Crisafulli, Giorgio Corti, Benedetta Mussolin, Federica Baldi, Michela Buscarino, Alice Bartolini, Emanuele Valtorta, Joana Vidal, Beatriz Bellosillo, Giovanni Germano, Filippo Pietrantonio, Agostino Ponzetti, Joan Albanell, Salvatore Siena, Andrea Sartore-Bianchi, Federica Di Nicolantonio, Clara Montagut, Alberto Bardelli

**Affiliations:** 1 Candiolo Cancer Institute—FPO, IRCCS, 10060 Candiolo, Torino, Italy; 2 FIRC Institute of Molecular Oncology (IFOM), 20139 Milano, Italy; 3 Department of Oncology, University of Torino, SP 142, Km 3.95, 10060 Candiolo, Torino, Italy; 4 Niguarda Cancer Center, Grande Ospedale Metropolitano Niguarda, 20162 Milano, Italy; 5 Medical Oncology Department, Hospital del Mar, 08003 Barcelona, Spain; 6 Cancer Research Program, FIMIM (Hospital del Mar Medical Research Institute), Hospital del Mar, 08003 Barcelona, Spain; 7 Pathology Department, Hospital del Mar, 08003 Barcelona, Spain; 8 Medical Oncology Department, Fondazione IRCCS Istituto Nazionale dei Tumori, 20133 Milano, Italy; 9 Colorectal Cancer Unit, Medical Oncology Division 1, San Giovanni Battista Hospital, 10133 Torino, Italy; 10 Dipartimento di Oncologia e Emato-Oncologia, Università degli Studi di Milano, 20122 Milano, Italy

## Abstract

Blockade of the epidermal growth factor receptor (EGFR) with the monoclonal antibodies cetuximab or panitumumab is effective in a subset of colorectal cancers (CRCs), but the emergence of resistance limits the efficacy of these therapeutic agents. At relapse, the majority of patients develop *RAS* mutations, while a subset acquires *EGFR* extracellular domain (ECD) mutations. Here we find that patients who experience greater and longer responses to EGFR blockade preferentially develop *EGFR* ECD mutations, while *RAS* mutations emerge more frequently in patients with smaller tumour shrinkage and shorter progression-free survival. In circulating cell-free tumour DNA of patients treated with anti-EGFR antibodies, *RAS* mutations emerge earlier than *EGFR* ECD variants. Subclonal *RAS* but not *EGFR* ECD mutations are present in CRC samples obtained before exposure to EGFR blockade. These data indicate that clonal evolution of drug-resistant cells is associated with the clinical outcome of CRC patients treated with anti-EGFR antibodies.

## Abstract (web summary)

Some colorectal cancer patients respond well to treatment with anti-EGFR antibodies, however response is almost invariably followed by acquired resistance. Here, the authors show that patients with shorter responses acquire *RAS* mutations, while those relapsing later preferentially develop mutations in the extracellular domain of *EGFR*.

Human tumours such as colorectal, lung and breast cancers are thought to evolve through expansion of clonal waves driven by the acquisition of genomic alterations^1^,^2^. Tumour progression is fuelled by genomic instability, which sustains the continuous emergence of new genetic variants that are then fixed in the population by selective pressures not yet completely understood^3^.

Genomic instability and clonal evolution result in widespread cellular heterogeneity, which allows human cancers to survive pressures exerted by drugs designed to target oncogenic dependencies^4^,^5^. The intrinsic ability of subclonal cell populations to evolve when challenged with anticancer agents is arguably the major limitation to further progress in the medical treatment of oncological patients.

In colorectal cancers the limitations imposed by the emergence of drug resistance are manifest during treatment with the anti-EGFR antibodies cetuximab or panitumumab. In this setting, remarkable clinical responses can be observed, which are invariably followed by tumour progression and relapse^6^,^7^. Acquired resistance to EGFR blockade is driven by the emergence of *KRAS/NRAS* mutations or the development of *EGFR* extracellular domain (ECD) variants, which impair antibody binding^8^,^9^. Studies in clinical specimens indicate that *RAS* mutations are detected more frequently than *EGFR* ECD mutations in samples of patients that relapse on EGFR blockade^10^,^11^,^12^. Considering that *EGFR* ECD mutations completely abrogate the interaction with anti-EGFR monoclonal antibodies, it is unclear why only a fraction of relapsed tumours display these alterations. Furthermore, whether and how the emergence of *RAS* or *EGFR* ECD mutations impacts the clinical development of patients receiving cetuximab or panitumumab is presently undefined. We find that clonal evolution during the acquisition of resistance impacts the clinical response to anti-EGFR therapy in colorectal cancer, and this may be influenced by the subclonal mutational landscape and environmental pressure on the tumour.

## Results

### Acquired *RAS* or *EGFR* mutations and anti-EGFR response

We considered whether the emergence of distinct resistance mechanisms might affect the clinical outcome of metastatic colorectal cancer (CRC) patients treated with anti-EGFR antibodies. To test this hypothesis, we examined the clinical characteristics of 27 individuals who initially responded but developed either *RAS* (*KRAS/NRAS*) or *EGFR* ECD mutations at progression during treatment with cetuximab or panitumumab (Table 1 and Supplementary Tables 1 and 2). No associations were found between mutations at acquired resistance (*RAS* versus *EGFR*) and clinico-pathological characteristics of the patients.

**Figure 1:**
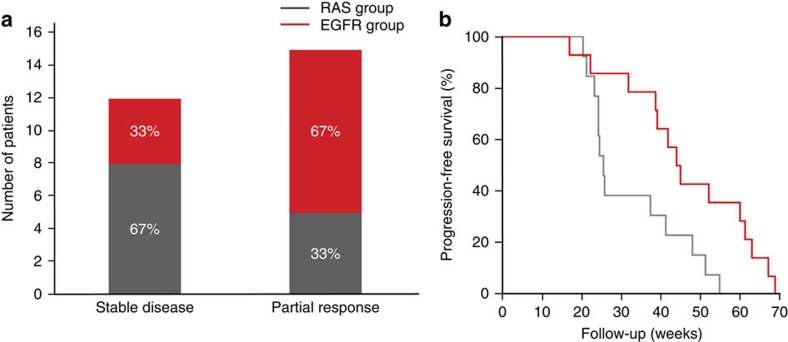
Clinical benefit of CRC patients treated with anti-EGFR therapy according to the emergence of *EGFR* ECD versus *RAS* mutations. (**a**) Response measured by RECIST criteria 1.1 in 27 CRC patients, according to the emergence of *EGFR* ECD versus *RAS* mutations detected in tumour tissue and/or plasma. Most patients with emergence of *EGFR* ECD mutations achieved a partial response, whereas patients with emergence of *RAS* mutations mostly had stable disease as best response (*P*=0.128 by Fisher's exact test). Cases with co-existence of *EGFR* ECD and *RAS* mutations were excluded from the group of *RAS* mutant tumours (RAS group) and included in the group of *EGFR* ECD mutant tumours (EGFR group). (**b**) Kaplan–Meier estimates of PFS according to the emergence of *RAS* mutations versus *EGFR* ECD mutations detected in tumour tissue and/or plasma. The hazard ratio for the RAS group (grey line) as compared with the EGFR group (red line) was 2.56 (95% CI, 1.06–6.21; *P*=0.013 by a stratified log-rank test). Median PFS time in the RAS group was 25.6 weeks (95% CI, 24–27), as compared with 44.6 weeks (95% CI, 38–49) in the EGFR group.

However, the mutational profile at the time of progression was correlated with the clinical outcome, defined as initial complete response, partial response or stable disease for more than 16 weeks, as assessed by Response Evaluation Criteria in Solid Tumors (RECIST) version 1.1. Mutations in *RAS* were detected in 20 patients and mutations in *EGFR* ECD in 14 patients. The co-occurrence of *RAS* and *EGFR* ECD mutations was detected in 7 of these cases (Table 1 and Supplementary Table 2). We found that the emergence of *RAS* or *EGFR* ECD mutations correlated with clinical response. Namely, most patients who achieved stable disease as best response developed *RAS* mutations, whereas patients with partial response preferentially showed *EGFR* ECD mutations, either with or without *RAS*, in their tumours (Fig. 1a). Most notably, a clear distinction was observed when progression-free survival (PFS) was used to stratify patients who developed only *RAS* or *EGFR ECD* mutations. Median PFS for the EGFR group was 44.6 weeks (95% confidence interval (CI), 38–49) versus 25.6 weeks (95% CI, 24–27) for the RAS group, hazard ratio 2.56 (95% CI, 1.06–6.21; *P*=0.013 by a stratified log-rank test) (Fig. 1b). These findings suggest that CRC patients who develop *RAS* mutations on EGFR blockade achieve reduced tumour shrinkage and shorter duration of response than patients in which *EGFR* ECD mutations emerge during therapy.

### *RAS* and *EGFR* dynamics in ctDNA during EGFR blockade

**Figure 2:**
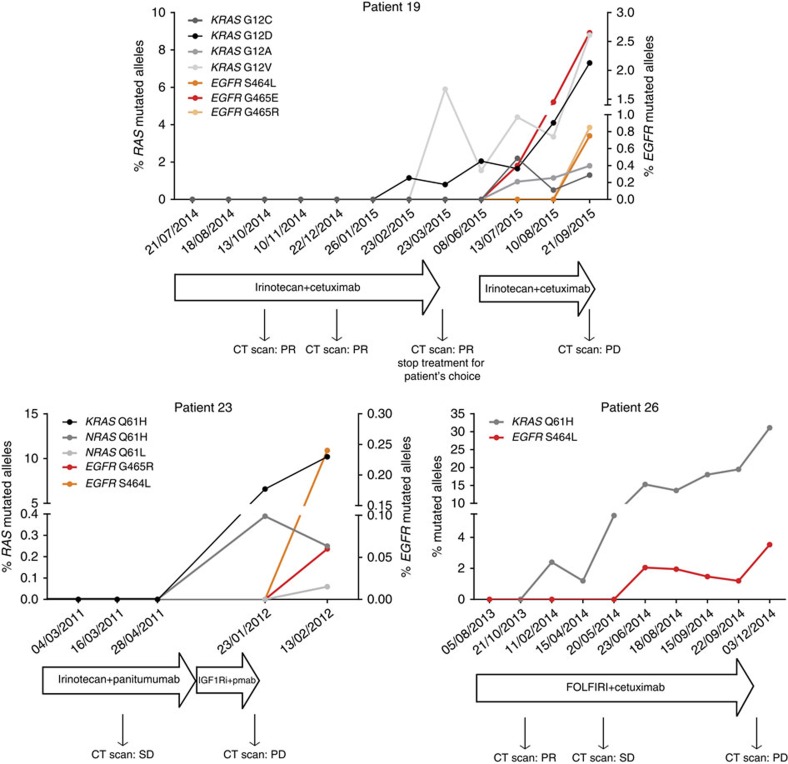
Longitudinal analysis of plasma ctDNA collected during anti-EGFR therapy. Shades of black and grey indicate frequency of circulating *RAS* mutant alleles; and shades of red and orange indicate frequency of circulating *EGFR* mutant alleles. All patients achieved partial response or disease stabilization under EGFR blockade administered alone or in combination with standard chemotherapy. *RAS* mutant clones are apparent in the circulation months earlier than *EGFR* clones in all of the three cases. CT, computed tomography; PD, progressive disease; Pmab, panitumumab; PR, partial response; SD, stable disease.

The clinical results described above suggest that *RAS* and *EGFR* ECD mutations are correlated with the extent of tumour shrinkage and duration of response in CRC patients treated with anti-EGFR antibodies. We postulated that this reflected the dynamics of clonal evolution of *RAS* and *EGFR* ECD mutant cells during treatment. To test this we studied the molecular profile of circulating cell-free tumour DNA (ctDNA), an approach we have previously exploited to identify and monitor resistance to targeted therapies in CRC patients^13^. We therefore analysed liquid biopsies of CRC patients who developed resistance to EGFR blockade. Within our clinical database, we identified three patients in which *RAS* and *EGFR *ECD mutations were concomitantly present in ctDNA collected at progression and for which longitudinal samples had been collected. Molecular profiling on blood draws obtained at different time points during treatment revealed that *RAS* mutant clones, which were undetectable when therapy was initiated, became evident earlier than *EGFR* ECD variants (Fig. 2). Owing to the limitations of liquid biopsies, it cannot be determined if the mutations occur in the same cell.

### Evolution of mutations in CRC cells during EGFR blockade

The differential impact of *RAS* and *EGFR* ECD mutations on PFS and the dynamics observed in liquid biopsies suggest that colorectal tumours evolve along distinct molecular trajectories according to the occurrence of specific resistance mutations. To gather insights into the molecular roots of these findings, we used two CRC cell models (LIM1215 and OXCO-2) both of which are sensitive to EGFR blockade and amenable to the analysis of individual subclones. LIM1215 and OXCO-2 cells were treated continuously with cetuximab for several months; at the end of the treatment, the presence of *RAS* and *EGFR* ECD mutations was assessed using droplet digital PCR (ddPCR). When compared with the parental (sensitive) counterpart, resistant cell populations displayed *KRAS*, *NRAS* or, less frequently, *EGFR* ECD mutations^11^ (Supplementary Fig. 1). To study the molecular characteristics of resistant clones, we performed limiting cell dilutions followed by DNA sequencing. This analysis retrieved clones carrying concomitantly both *NRAS* and *EGFR* ECD mutations (Supplementary Fig. 1).

**Figure 3:**
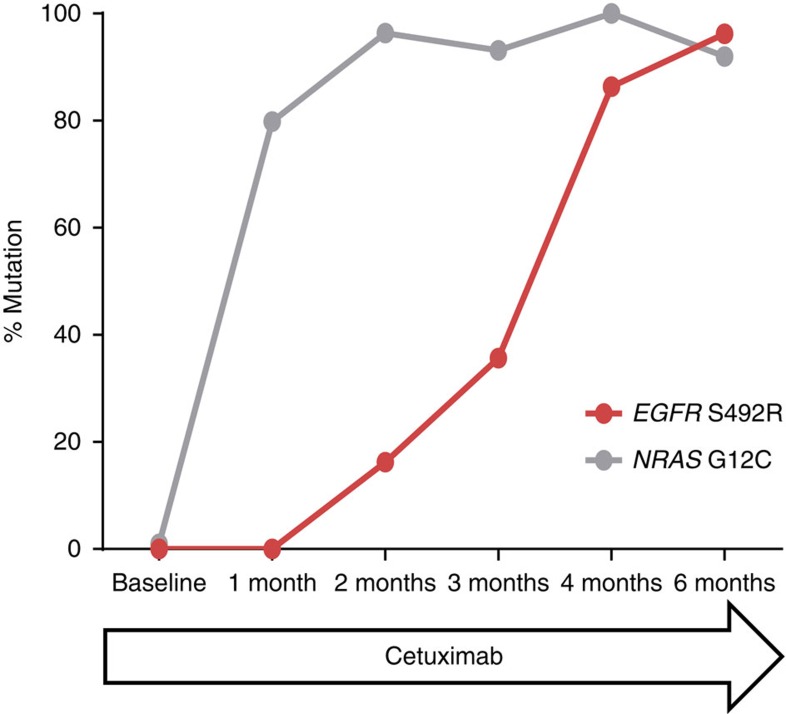
Selection of *RAS* and *EGFR* ECD mutations during development of resistance to EGFR blockade in CRC cells. Mutational profiles were used to monitor clonal dynamics of LIM1215 CRC cells under cetuximab treatment. Frequency of the *NRAS* G12C and *EGFR* S492R mutations at each time point are shown as determined by NGS. The frequencies presented were adjusted for gene copy number as it was determined by FISH analysis that LIM1215 has a duplication of the *EGFR* locus (7p.11.2) in the p-arm of chromosome 7 (Supplementary Fig. 5).

To gain further insights into the clonal dynamics leading to the expansion of clones carrying single or multiple resistance alleles, LIM1215 cells were genetically tracked with a lentiviral library of ∼50 million unique molecular barcodes consisting of two 18 base pair (bp) random DNA sequences (Supplementary Fig. 2). The progeny of individually barcoded cells can be later identified by the corresponding barcode sequence. The primer region flanking the barcode sequence allows for the amplification of the barcode from genomic DNA (gDNA) that can then be deconvoluted using next-generation sequencing (NGS) analysis^14^,^15^ (Supplementary Table 3). A barcoded population of one million cells was treated with cetuximab for ∼6 months. An equal number of cells were set aside for gDNA analysis as the representative baseline. As the cells underwent treatment with the anti-EGFR antibody, samples of the cell populations were collected at regular intervals and, at the end, all samples were analysed (Fig. 3). This analysis revealed that a clone with an *NRAS* G12C mutation rapidly became predominant in the population while the heterogeneity decreased as indicated by barcode analysis in Supplementary Fig. 3. After 2 months, another clone, labelled with a different barcode and carrying the *EGFR* S492R mutation, started to emerge. Limiting dilution assays showed that this clone carried both the *NRAS* G12C and the *EGFR* S492R mutation (Supplementary Fig. 3). The *EGFR* variant clone increased in subsequent time points, and when the experiment was terminated (6 months from start), the *EGFR* S492R mutant clone became dominant (Fig. 3 and Supplementary Fig. 3).

### Fitness analysis of *RAS* and *EGFR* ECD mutant clones

**Figure 4:**
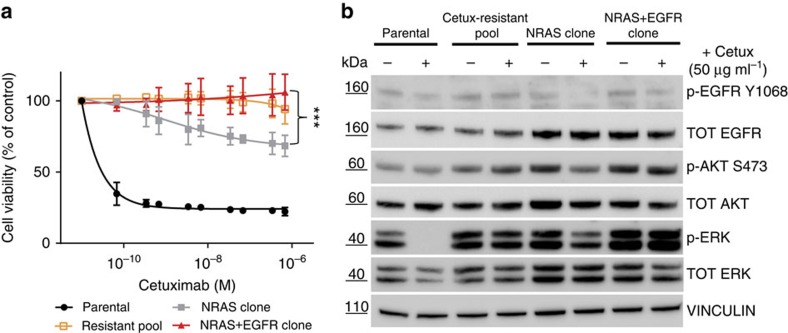
Fitness of individual and double-mutated cells in the presence of cetuximab. (**a**) Sensitivity to cetuximab of LIM1215 parental, resistant pool and single-cell clones containing the indicated mutations. Cell populations and individual clones were treated with increasing doses of cetuximab for 6 days. Cell viability was measured by ATP assay and the mean calculated as % of untreated control. Error bars are s.d. of three independent experiments with three technical replicates. ***P<0.001 by unpaired two-tailed Student's *t*-test (NRAS+EGFR clone versus NRAS clone). (**b**) Western blot analysis of the indicated cells treated with 50 μg ml^−1^ of cetuximab and lysed after 2 h of treatment. Lysates were immunoblotted with the indicated antibodies. Vinculin was included as a loading control. Cetux, cetuximab.

To provide mechanistic support for these data, drug sensitivity measurements were performed on cell populations and clones carrying the *NRAS* only or *EGFR* mutations. The results indicate that cells harbouring mutant *EGFR* had increased fitness as compared with the single (*NRAS*) mutant cells when drug pressure was applied (Fig. 4a). To further corroborate these findings, we performed clonogenic assays in the presence of cetuximab that confirmed that the *EGFR* mutant cells displayed increased fitness when challenged with anti-EGFR antibodies (Supplementary Fig. 4). To understand how the presence of single or dual mutations impacted the EGFR–RAS signalling pathway, we performed biochemical analyses. These experiments revealed that in the presence of cetuximab, ERK activation (measured by phosphorylation) is partially reduced in *NRAS* mutant cells as compared with untreated cells; while in cells carrying both *NRAS* and *EGFR* mutations, ERK activation is not affected by the addition of cetuximab (Fig. 4b). This is likely related to the inability of cetuximab to bind the receptor. These results indicate that the occurrence of *NRAS* mutations alone or in combination with *EGFR* ECD mutations differentially affects the EGFR-RAS signalling pathway.

### The subclonal landscape of CRC affects drug resistance

The data described in Table 1 and Fig. 1 suggest that the pre-existence of subclones carrying *RAS* but not *EGFR* ECD mutations affects the clinical outcome of CRC patients treated with cetuximab or panitumumab. Furthermore, liquid biopsies and functional analyses of cell populations support the possibility that *RAS* mutant clones emerge before *EGFR* ECD variants. We reasoned that these findings might be mechanistically explained if cells carrying *RAS* mutations were latently present in colorectal tumours before exposition to EGFR blockade. Specifically, we hypothesized that RAS pathway mutations (but not *EGFR* ECD variants) might emerge in colorectal tumours in response to ‘environmental' stress. We therefore contemplated which conditions might affect the emergence of *RAS* mutant subclones in otherwise wild-type (WT) CRC cell populations.

**Figure 5:**
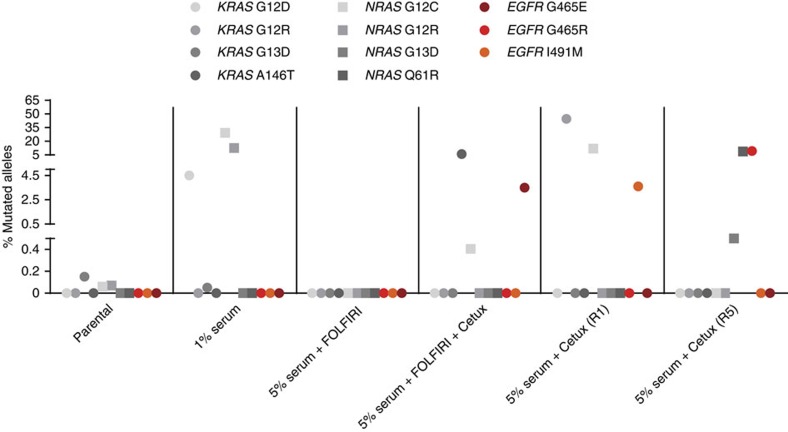
Emergence of *RAS* and *EGFR* ECD mutations in CRC cell populations. *RAS* and *EGFR* mutation frequencies were measured by ddPCR and are indicated for LIM1215 cells cultured in low-serum, chemotherapy, chemotherapy plus cetuximab and cetuximab-only conditions. R1 and R5 cetux indicate two resistant populations of LIM1215 generated independently. Cetux, cetuximab.

We previously reported that CRC cells sensitive to EGFR blockade are dependent on EGFR ligands^16^. We therefore postulated that reduced access to growth factors and nutrients may represent a stress condition leading to the emergence of RAS pathway mutations^17^,^18^. Moreover, as patients often receive anti-EGFR antibodies after or concomitantly with chemotherapy^19^, we wanted to assess if this could affect the clonal composition of colorectal tumours. To test these possibilities, we used LIM1215 cells. As previously reported, high-sensitivity genotyping using ddPCR detected very low levels of *RAS* mutations in LIM1215 cells that were never exposed to anti-EGFR antibodies (Fig. 5 and ref. 20). Notably, the same approach did not reveal the presence of *EGFR* ECD mutations (Fig. 5), even when an average sensitivity of 0.005% was achieved (Supplementary Table 4). These results are aligned with our previous data demonstrating that in both microsatellite stable (MSS) and microsatellite instability (MSI) CRC cell lines *EGFR* ECD mutations are never detected before administration of anti-EGFR treatment^11^.

To corroborate our observations, when sufficient material was available, a more sensitive molecular analysis was performed also on patients' tissue samples obtained at baseline. All patient samples were *RAS* WT using the standard of care assays; however, using a sensitive NGS-based technique, it was found that four of the patients indeed had pre-existing *RAS* mutations (Supplementary Table 1). These mutations were confirmed by competitive allele-specific TaqMan PCR. Notably, the same mutations detected at baseline were also detected at progression (Supplementary Tables 1 and 2). No *EGFR* ECD mutations were detected at baseline. The clinical and preclinical results are consistent with other studies of CRC tumour samples from patients that were never exposed to anti-EGFR antibodies as it has been demonstrated that they may carry subclonal *RAS* mutations but not pre-existing *EGFR* ECD mutations^10^,^12^,^21^.

We then cultured LIM1215 CRC cells in the presence of reduced serum conditions (1% fetal bovine serum; FBS) for 4 months and used ddPCR to measure the presence of *RAS* and *EGFR* mutations. Notably, when LIM1215 cells were cultured in low-serum conditions, the levels of *KRAS* and *NRAS* mutations increased, while *EGFR* ECD mutations were not detected (Fig. 5). While MSI cellular models have inherent limitations, they often recapitulate the mechanisms of resistance to targeted therapy that were observed in clinical specimens. For example, the emergence of *RAS* mutations on EGFR blockade in CRC patients was correctly predicted by experiments performed in MSI cell lines as we previously reported^20^. Furthermore, subclonal *RAS* mutant cells were detected in the LIM1215 cell line before anti-EGFR treatment similarly to what has been found in patients that were never exposed to anti-EGFR antibodies^12^.

To test whether chemotherapy affected the emergence of *RAS* or *EGFR* ECD mutations, we treated LIM1215 cells with FOLFIRI (5FU+irinotecan) alone or in combination with cetuximab. To parallel the chemotherapy schedule administered to patients, cells were treated with FOLFIRI for four cycles of 2 days each (each cycle was repeated every 2 weeks and was separated from the next one by 12 days). At the end of the fourth cycle of chemotherapy, cells were allowed to repopulate in the absence of drugs, and growing cells were then collected. *RAS* or *EGFR* ECD mutations did not emerge in cells treated with FOLFIRI alone, while the FOLFIRI+cetuximab-treated cells displayed *RAS* and *EGFR* ECD mutations, as also observed in cells treated with cetuximab alone (LIM1215 R1 and R5) (Fig. 5). These results are in concordance with what has been observed in patient samples regarding the lack of emergence of *KRAS* mutations after chemotherapy treatment^20^.

## Discussion

The emergence of drug resistance is arguably the major limitation to the development of effective targeted therapies. Genes encoding for protein and lipid kinases are frequently altered in cancer and can be pharmacologically inhibited. Accordingly, a large number of kinases have been targeted with remarkable, albeit often transitory, effect in several cancer types^22^. In the last 10 years, the molecular mechanisms of resistance to kinase inhibitors have been elucidated in details in multiple tumour types. Generally, clones carrying mutations or other genetic alterations, which confer an advantage in the presence of the inhibitor, drive resistance. The innate genomic instability of cancers can contribute to this process^23^. For example, acquired mutations in the catalytic domain of the receptor tyrosine kinase *cKIT* limit the effectiveness of the kinase inhibitor imatinib in gastrointestinal stromal tumours^24^. In other settings, resistance can arise not only through alterations that affect the drug target but also through activation of alternative pathways^8^,^25^. For example, the *EGFR* T790M variant emerges frequently in NSCLC carrying *EGFR* mutations that are treated with erlotinib or gefitinib^26^. However, lung cancer cells can also become resistant by constitutive activation of parallel pathways exemplified by *MET* amplification^27^. Similarly, colorectal tumours that are treated with anti-EGFR antibodies develop resistance through three main mechanisms: mutations in the antibody-binding site (such as the *EGFR* ECD variants)^10^,^11^; activation of alternative pathways^28^,^29^; or reactivation of downstream signalling (such as *KRAS* or *NRAS* mutations)^30^. As mentioned above, the acquisition of drug-resistance mutations can be affected by the genomic instability of cancer. For example, in colorectal cancers, MSI can impact the acquisition of mutations^31^,^32^. New information is emerging suggesting that gDNA containing oncogenic mutations, which can also contribute to resistance, can be found in exosomes^33^,^34^ and these could be the means for horizontal mutant gene transfer^35^.

Using CRC as a model system, we report that—from a clinical perspective—mutations that confer resistance to EGFR blockade are not created equal. Notably, we provide evidence that patients who experience more profound and longer responses to EGFR blockade preferentially develop *EGFR* ECD mutations, while *RAS* mutations emerge more frequently in patients with limited tumour shrinkage and shorter PFS. Intrigued by these clinical observations, we exploited populations of cancer cells to study clonal evolution during EGFR blockade. Our findings support a model in which RAS pathway mutations are selected under distinct environmental ‘stress' (such as reduced access to growth factors and nutrients, but not to chemotherapy agents) as they confer a growth advantage in these conditions. Under the same circumstances, *EGFR* ECD mutations do not provide a survival advantage and are positively selected only when the cells are challenged with anti-EGFR antibodies. These results, albeit limited to the cell lines we studied, support a model whereby cells carrying *RAS* mutations are latently present (that is, pre-exist) in WT CRC populations sensitive to EGFR blockade and rapidly expand under anti-EGFR drug pressure.

Clinically, this could translate into smaller tumour shrinkage and earlier progression on anti-EGFR treatment in patients. Differently, *EGFR* ECD mutant subclones do not pre-exist in WT CRC but emerge during drug-driven tumour evolution and confer a fitness advantage throughout anti-EGFR treatment. This translates into more profound tumour responses and longer time to anti-EGFR treatment failure in patients who preferentially develop *EGFR* ECD mutations. These results are aligned with previous reports that CRC cell lines may contain pre-existing but low levels of *RAS* mutations, but they can also acquire mutations *de novo*^20^. This would apply to the acquisition of mutations in the *EGFR* ECD during anti-EGFR therapy as there have been no reports of these mutations pre-existing in cell lines or patients before anti-EGFR therapy^10^,^11^,^12^,^21^,^36^.

Although the analysis of clinical samples was retrospective, the results are intriguing and provide an interesting hypothesis for further studies. Overall, our data suggest that in populations of CRC cells, resistance to EGFR blockade is accompanied by multiple waves of clonal expansion and can ultimately lead to the emergence of clones carrying multiple mutations. This process is reminiscent of the multistep progression model associated with the clinical evolution of colorectal tumours. Accordingly, we propose that ‘environmental' and ‘drug' pressures analogously affect the genetic evolution of CRC cells.

## Methods

### Patient samples and clinical characteristics

We included patients with histologically confirmed metastatic CRC treated with anti-EGFR-based therapy recruited at Hospital del Mar (Barcelona, Spain) between January 2013 and January 2016, Ospedale Niguarda (Milano, Italy) between March 2011 and May 2014, Fondazione IRCCS Istituto Nazionale dei Tumori (Milano, Italy) between July 2012 and October 2013, and Città della Salute e della Scienza, San Giovanni Battista Hospital (Torino, Italy) between August and October 2013. Protocols through which tumour specimens were obtained were the following: Hospital del Mar (protocol CEIC-2012/4741/I); Ospedale Niguarda (protocols 1014/09 and 194/2010); Fondazione IRCCS Istituto Nazionale dei Tumori (protocol INT 73/12); and Città della Salute e della Scienza, San Giovanni Battista Hospital (protocol PROFILING). All protocols were approved by the local Ethics Committee of each institution. All participating patients signed written informed consent. All included patients had acquired resistance to anti-EGFR therapy defined as disease progression following (i) complete response or partial response or (ii) stable disease for more than 16 weeks, and at the time of progression, a *RAS* (*KRAS+NRAS*) and/or *EGFR* mutation was detected. Response was evaluated according to the RECIST version 1.1 (ref. 37). PFS was defined as the time from anti-EGFR treatment start to disease progression or death. One patient (#23) received a first course of 6 months with anti-EGFR and irinotecan, and a subsequent course of 6 weeks with anti-EGFR in combination with an insulin-like growth factor receptor type I inhibitor. Concerning Hospital del Mar working group, biological samples were obtained from Parc de Salut Mar Biobank (MARBiobanc). Baseline pretreatment mutation analysis in tissue (primary tumour or metastasis) was carried out using the standard of care procedures for patients with mCRC considered for anti-EGFR treatment under the local direction of the clinical Institutions that participated in the study. Standard Sanger sequencing and pyrosequencing were used for pretreatment mutation analyses. Hospital del Mar used the therascreen *RAS* RGQ PCR Kit (Qiagen, Ref 874011) and *RAS* Extension Pyro Kit (Qiagen, Ref 971590). San Giovanni Battista Hospital used the PYROMARK Q96 ID kit (Diatech Pharmacogenetics, cod. UP032). Ospedale Niguarda and IRCCS Istituto Nazionale dei Tumori used Sanger Sequencing according to the protocol described in refs 38, 39.

For very-high-sensitivity pretreatment analyses, *RAS* and *EGFR* mutations at baseline were analysed in tissue samples. Mutations in exons 2, 3 and 4 from *KRAS* and *NRAS* and exon 12 of *EGFR* were analysed by pyrosequencing in the NGS 454 GS Junior platform (Roche Applied Science). Library preparation was performed with the ONCOGENBASIC-S1 KIT (Seqplexing, Valencia, Spain) to simultaneously amplify *BRAF* (exon 15), *KRAS* (exons 2, 3 and 4) and *NRAS* (exons 2, 3 and 4). To analyse *EGFR* ECD, two amplicons were generated using two primer sets (Supplementary Table 3). Processed and quality-filtered reads were analysed using the GS Amplicon Variant Analyzer software version 2.5p1 (Roche). Mutations detected by NGS were confirmed by competitive allele-specific TaqMan PCR (CAST-PCR, Applied Biosystems Ref 4465804). The following individual assays were used: *KRAS* p.Q61H Hs00000137_mu; *KRAS* p.Q61L Hs00000135_mu; *NRAS*_p.G12S—Hs00000793_mu; and *NRAS*_p.Q61K—Hs00000804_mu. All samples were also screened by the PGM Ion Torrent platform using the OncoMine Focus 318 DNA Assay (Life Technologies, Ref A28548G) to prepare the libraries.

*RAS* and *EGFR* mutations at the time of progression were analysed in both tissue and plasma samples. *RAS* mutations in tissue samples were determined by the Therascreen RAS RGQ PCR KIT for *KRAS* codons 12 and 13, and RAS Extension Pyro Kit (Qiagen) for *KRAS* exons 3 and 4 and *NRAS* (exons 2, 3 and 4) as described above. *EGFR* exon 12 in tissue samples was analysed by Sanger sequencing to uncover *EGFR* ECD mutations as described previously^11^. Plasma samples only were obtained from Ospedale Niguarda, Città della Salute e della Scienza—San Giovanni Battista Hospital and Fondazione IRCCS Istituto Nazionale dei Tumori, and assessed by ddPCR as described in the Methods section.

### Automated ctDNA isolation from plasma samples

ctDNA was extracted from 1 ml plasma using the Maxwell RSC ccfDNA Plasma Kit with the automated Maxwell RSC Instrument (Promega) according to the manufacturer's instructions.

### Lentivirus packaging

The lentiviral barcode library was packaged in HEK-293T cells using 10 μg of the Cellecta 13K × 13K barcode library plasmid and second-generation packaging vectors pCMV-dR8.74 (6.5 μg), pRSV-REV (2.5 μg) and pMD2-VSV-G (3.5 μg) per 10 cm dish (10 dishes total) following the standard protocol for transfection by calcium phosphate precipitation^40^. Media were replaced after 16 h, DNAse treated (1 U ml^−1^) 24 h post replacement and collected/filtered 24 h later for virus collection.

### Cell models

The LIM1215 parental cell line^41^ was obtained from Prof Robert Whitehead, Vanderbilt University, Nashville, with permission from the Ludwig Institute for Cancer Research Ltd (New York, NY) and were cultured in RPMI-1640 medium (Invitrogen) supplemented with 10% FBS and insulin (1 μg ml^−1^); OXCO-2 cell lines were a kind gift from Dr V. Cerundolo in March 2010 (Weatherall Institute of Molecular Medicine, University of Oxford, UK), and were cultured in Iscove's medium (Invitrogen) supplemented with 10% FBS. Both LIM1215 and OXCO-2 are mismatch repair deficient (microsatellite unstable) as confirmed by the MSI Analysis System, Version 1.2 kit (Promega). The authentication of each cell line was completed by Cell ID System and by Gene Print 10 System (Promega), through short tandem repeats (STR) at 10 different loci (D5S818, D13S317, D7S820, D16S539, D21S11, vWA, TH01, TPOX, CSF1PO and amelogenin). Amplicons from multiplex PCR products were separated by capillary electrophoresis (3730 DNA Analyzer, Applied Biosystems) and analysed using GeneMapperID software from Life Technologies. These STR profiles were cross-validated with the available STR from other cell bank databases. All cell lines were tested and resulted negative for mycoplasma contamination with Venor GeM Classic kit (Minerva Biolabs).

### Generation of barcoded founder populations

LIM1215 parental CRC cells were used to make the founder population. In all, 1 × 10^6^ cells were transduced at a multiplicity of infection of 0.1 with 5 μg ml^−1^ polybrene. Media were changed 24 h post transduction. Puromycin (1 μg ml^−1^) was added at 96 h post transduction to select cells with lentiviral integration and continued for 96 h.

### Serum-deprived and drug-treated cells

For the serum deprivation experiment, the barcode founder LIM1215 cells were cultured in RPMI supplemented with 1% FBS for 4 months. Cells were collected at 4 months for preparation of gDNA. For chemotherapy experiments, LIM1215 cells were cultured in their respective media supplemented with 5% serum and treated with FOLFIRI (10 μM 5FU+100 nM SN-38, active metabolite of irinotecan) alone or in combination with cetuximab (350 nM). To parallel the chemotherapy schedule administered to human patients, cells were treated with FOLFIRI for four cycles of 2 days each; each cycle is separated from the next one by 12 days. At the end of the fourth cycle of chemotherapy, cells were allowed to repopulate in absence of drugs, and growing cells were then collected (total: 2.5 months). Instead, in combinatorial FOLFIRI+cetuximab setting, cetuximab was continuously maintained throughout the treatment period until a resistant population emerged (5 months). gDNA was extracted using the ReliaPrep gDNA Tissue Miniprep System (Promega).

LIM1215 and OXCO-2 cetuximab-resistant cells were described previously^11^,^20^,^30^. gDNA was extracted using the Wizard SV Genomic DNA Purification System (Promega). For generation of barcoded cetuximab-resistant cells, the barcode founder population was treated continuously with 350 nM cetuximab in their media supplemented with 5% serum for ∼6 months. Cells were screened for resistance to cetuximab starting at ∼1 month and at 1-month increments until resistance was achieved. Cells were saved for gDNA analysis and aliquots were preserved in liquid nitrogen at each screening.

For limiting cell dilution, the resistant pools were diluted such that they are plated at 1 cell per well (5 cells per ml) in a 96-well tissue culture plate. Wells are screened for single colonies, colonies consolidated, and screened for barcodes and/or mutations. gDNA was extracted from limiting cell dilution clones using Wizard SV 96 Genomic DNA Purification System (Promega) and were analysed for the barcode sequence and/or mutations in *KRAS* (exons 2, 3 and 4), *NRAS* (exons 2 and 3) and *EGFR* (exon 12) with Sanger sequencing using automated sequencing by ABI Prism 3730 (Applied Biosystems). Primer sequences for *KRAS*, *NRAS* and *EGFR* have been described previously^11^,^20^,^30^. The barcode primer sequences were designed by Cellecta based on the lentiviral vector cassette and are listed in Supplementary Table 3.

### Fluorescence *in situ* hybridization analysis

To measure *EGFR* gene copy numbers, dual-colour fluorescence *in situ* hybridization (FISH) analysis was performed as previously described^20^. More in details: dual-colour FISH analysis was performed on metaphase chromosomes and interphase nuclei obtained from the parental and resistant clones of LIM1215 following the standard procedures. To identify possible alterations in *EGFR* gene copy number, EGFR(7p12)/CEN7q(7q11.21) probes (Abnova, Catalog Number FG0005) labelled, respectively, with Texas Red and fluorescein isothiocyanate were used. Probes and target DNA were co-denatured for 5 min at 75 °C and then hybridized overnight at 37 °C. Slides were washed with post-hybridization buffer (DakoGlostrup) at 73 °C for 5 min and counterstained with 4,6-diamino-2-phenylindole (DAPI II; Vysis). FISH signals were evaluated with a Zeiss Axioscope Imager. Z1 (Zeiss) equipped with single and triple band pass filters. Images for documentation were captured with a charge-coupled device camera and processed using the MetaSystems Isis software.

### Barcode NGS and analysis

gDNA collected from the barcoded cells during the cetuximab resistance protocol were prepared using phenol:chloroform extraction and ethanol precipitation. The extracted DNA was quantitated using the Qubit dsDNA BR Assay (Invitrogen). A unit of 150 ng of gDNA was used to amplify the barcode sequence and generate the amplicon library using Phusion High-Fidelity DNA Polymerase (New England Biolabs). For the cetuximab resistance experiments, the primers (HPLC purified) used were generated from the lentiviral vector cassette sequence and contain the adaptors specific for 454 GS FLX Titanium Lib-L sequencing system (Roche) (Supplementary Table 3). The forward primer contains a unique multiplex identifier allowing samples to be pooled for the sequencing reaction and sorted post sequencing. The PCR products were size selected with the E-Gel Size Select Gels (Life Technologies) and purified twice using Agencourt AMPure XP as described in the 454 Sequencing Amplicon Library Preparation Method Manual (Roche Diagnostics). Purified products were screened for quality using the 2100 Bioanalyzer with the High Sensitivity DNA kit (Agilent Technologies) and quantitated using the Qubit dsDNA HS Assay (Invitrogen). Individual amplicons were diluted to 1 × 10^9^ molecules per μl, then samples to be sequenced together were pooled (five samples per pool) and diluted to 1 × 10^7^ molecules per μl to generate the amplicon library. The library was amplified and prepared for sequencing using the emulsion PCR large volume kit for Lib-L. Two libraries containing five samples each were loaded into two lanes of the PicoTiter Plate as described in the Sequencing Method Manual (Roche Diagnostics).

Analysis was performed considering that every sequence should include a linear structure of two 32 bp-long primers surrounding the 40 bp random barcode. We used BLAT^42^ to identify the position of this structure and applied a custom script to extract it for further analysis; reads that did not match this sequence were discarded. To lower the noise due to sequencing error, we evaluated the distance between every barcode by applying an all-against-all BLAST^43^ alignment. We considered as the same barcode those sequences displaying no more than one mismatch with each other. Finally, we counted the occurrence of each barcode sequence.

### Mutational profile by NGS

Libraries were prepared with Nextera Rapid Capture Custom Enrichment Kit (Illumina Inc), according to the manufacturer's protocol. Preparation of libraries was performed starting from 100 ng of gDNA. gDNA was fragmented using transposones, adding simultaneously adapter sequences. Purified tagmented gDNA was used as template for subsequent PCR to introduce unique sample indexes. The size distribution of the DNA fragments was assessed using the 2100 Bioanalyzer with the High Sensitivity DNA assay kit (Agilent Technologies). Equal amounts of DNA libraries were pooled and subjected to targeted panel hybridization capture as previously described^13^. Libraries were then sequenced using the Illumina MiSeq sequencer (Illumina Inc.).

FASTQ files generated by Illumina MiSeq were preprocessed to remove adapter sequences and bases with a Phred quality score <20. They were mapped to the human reference, assembly hg19, using BWA-mem algorithm^44^. PCR duplicates were then removed using the RMDUP command of SAMtools package^45^. We used a custom pipeline for NGS, to call somatic variations when supported by at least 1% allelic frequency and 5% Fisher's test significance level. Mutational discovery analyses were performed according to previously published methods^13^ comparing each of the different time points with parental samples. Mutations were annotated by a custom script printing out gene information, number of normal and mutated reads, the allelic frequencies and the variation effect.

### Droplet digital PCR analysis

Plasma circulating ctDNA from CRC patients and gDNA from CRC cells were amplified using ddPCR Supermix for Probes (Bio-Rad) using KRAS, NRAS (PrimePCR ddPCR Mutation Assay, Bio-Rad) and EGFR (custom-designed assays) for point mutations. Catalogue numbers and custom-designed probe sequences are indicated in Supplementary Table 5. ddPCR was then performed according to the manufacturer's protocol and the results reported as percentage or fractional abundance of mutant DNA alleles to total (mutant plus WT) DNA alleles. A volume of 8–10 μl of DNA template was added to 10 μl of ddPCR Supermix for Probes (Bio-Rad) and 2 μl of the primer/probe mixture. Droplets were generated using the Automated Droplet Generator (Auto-DG, Bio-Rad), where the reaction mix was added together with Droplet Generation Oil for Probes (Bio-Rad). Droplets were then transferred to a 96-well plate (Eppendorf) and then thermal cycled with the following conditions: 5 min at 95 °C; 40 cycles of 94 °C for 30 s; 55 °C for 1 min followed by 98 °C for 10 min (Ramp Rate 2 °C s^−1^). Droplets were analysed with the QX200 Droplet Reader (Bio-Rad) for fluorescent measurement of FAM and HEX probes. Gating was performed based on positive and negative controls, and mutant populations were identified. The ddPCR data were analysed with QuantaSoft analysis software (Bio-Rad) to obtain fractional abundance of the mutant DNA alleles in the WT/normal background. The quantification of the target molecule was presented as number of total copies (mutant plus WT) per sample in each reaction. Fractional abundance is calculated as follows: FA %=((Nmut/(Nmut+Nwt)) × 100), where Nmut is number of mutant events and Nwt is number of WT events per reaction. ddPCR analysis of normal control (from cell lines) and no DNA template controls were always included. Samples with positive events that were too low were repeated at least twice in independent experiments to validate the obtained results. Sensitivity is calculated as follows: sensitivity=((1/(*C*/2)) × 100, where *C* is the number of total target copies per ddPCR reaction (mutant plus WT). The number of mutated events required to be detected to call a sample positive for that particular target is two. If the fractional abundance from those two events is below the calculated sensitivity in the reaction, then it is a false positive.

### Cell viability assays

Cetuximab was obtained from the Pharmacy at Grande Ospedale Metropolitano Niguarda, Milan, Italy. LIM1215 cells were seeded (2 × 10^3^ per well) in 100 μl medium in 96-well culture plates. After 24 h, 100 μl of a serial dilution of cetuximab was added to the cells, and medium-only wells were included as controls. Plates were incubated at 37 °C in 5% CO_2_ for 6 days, after which cell viability was assessed by ATP content using the Cell Titer-Glo Luminescent Assay (Promega). Measurements were recorded using the Spark Microplate Reader (Tecan). Treated wells were normalized to untreated. Data points represent mean±s.d. of three independent experiments performed with three technical replicates.

### Clonogenic assays

LIM1215 cells were seeded (4 × 10^3^ per well in 24-well culture plates). After 24 h, a serial dilution of cetuximab was added to the cells, and medium-only in control wells. Four technical replicates were plated per experiment. Plates were incubated at 37 °C in 5% CO_2_ for 8 days, after which cells were fixed with 4% paraformaldehyde and stained with 1% crystal violet-methanol solution (Sigma-Aldrich).

### Immunoblot analysis

Total cellular proteins were extracted by solubilizing the cells in cold EB buffer (50 mM HEPES (pH 7.4), 150 mM NaCl, 1% Triton X-100, 10% glycerol, 5 mM EDTA and 2 mM EGTA; all reagents were from Sigma-Aldrich, except for Triton X-100 from Fluka) in the presence of 1 mM sodium orthovanadate, 100 mM sodium fluoride and a mixture of protease inhibitors (pepstatin, leupeptin, aprotinin, soybean trypsin inhibitor and phenylmethylsulfonyl fluoride). Extracts were clarified by centrifugation, and protein concentration was determined using BCA protein assay reagent kit (Thermo). Western blot detection was performed with enhanced chemiluminescence system (GE Healthcare) and peroxidase-conjugated secondary antibodies (Amersham). Chemoluminescent signal was acquired by the LAS4000 Image Reader (Fujifilm). The following primary antibodies were used for western blotting (all from Cell Signaling Technology, except where indicated): anti-phospho p44/42 ERK (Thr202/Tyr204); anti-p44/42 ERK; anti-phospho-AKT (Ser473); anti-AKT; anti-phospho EGFR (Tyr1068, Abcam); anti-EGFR (clone13G8, Enzo Life Sciences); and anti-vinculin (Sigma-Aldrich). All the antibodies were diluted 1:1,000 except for total EGFR (1:100) and vinculin (1:2,000). Full-length blot can be viewed in Supplementary Fig. 6.

### Statistical analysis

In patients, Fisher's exact test was used to assess the correlation between *RAS*/*EGFR* mutations and response to treatment. *χ*^2^-analysis and the Cox proportional regression model were used for survival analyses. Hazard ratios and 95% CIs were calculated. We estimated survival curves using the Kaplan–Meier method. We analysed PFS after anti-EGFR treatment according to *EGFR* mutations (including cases with co-existing *RAS* mutations) and *RAS* mutations (excluding cases with co-existing *EGFR* mutations). Patients for which the radiologic data were not available were censured at the last follow-up. All statistical tests included are appropriate and the assumption of normality for the group is accepted with a Shapiro–Wilk test of 0.94 (*P*=0.55). The variances are similar with a Levene value of *F*=0.772 (*P*=0.388). All the statistical tests were conducted at the two-sided 0.05 level of significance. Statistical analysis was performed with the SPSS Statistical Software (SPSS, Inc., Chicago, IL, USA).

Statistical analysis of the cell viability assays was performed using the software GraphPad PRISM 6.0. The *P* value was calculated by unpaired two-tailed Student's *t*-test. All values reported in the proliferation assays correspond to means±s.d. of at least three independent experiments, each with three experimental replicates.

### Data availability

NGS data for this study have been deposited in the European Nucleotide Archive (ENA) with the accession code PRJEB15863. The remaining data are available within the article and its Supplementary Information file, or available from the authors upon request.

## Additional information

**How to cite this article:** Van Emburgh, B. O. *et al*. Acquired *RAS* or *EGFR* mutations and duration of response to EGFR blockade in colorectal cancer. *Nat. Commun.* **7**, 13665 doi: 10.1038/ncomms13665 (2016).

**Publisher's note:** Springer Nature remains neutral with regard to jurisdictional claims in published maps and institutional affiliations.

## Supplementary Material

Supplementary Information Supplementary Figures 1-6 and Supplementary Tables 1-5.

## Figures and Tables

**Table 1:** Clinical characteristics of colorectal cancer patients with secondary resistance to anti-EGFR based therapy and emergence of *RAS* and/or *EGFR* ECD mutations.

	**All (*****N*****=27)**	***EGFR***** only (*****N*****=7)**	***RAS*** **only (*****N*****=13)**	***EGFR*****+*****RAS (N*****=7)**
Male sex (%)	13 (48)	5 (71)	6 (46)	2 (29)
Age (median, range)	60 (31–81)	64 (44–78)	59 (42–81)	55 (31–78)
*Primary tumour site*				
Right	5 (18%)	2 (29%)	1 (8%)	2 (29%)
Left and rectum	21 (78%)	4 (57%)	12 (92%)	5 (71%)
Unknown	1 (4%)	1 (14%)	0	0
*Anti-EGFR drug*				
Cetuximab	23 (85%)	6 (86%)	11 (85%)	6 (86%)
Panitumumab	4 (15%)	1 (14%)	2 (15%)	1 (14%)
*Chemotherapy*				
Irinotecan-based	20 (74%)	4 (57%)	10 (77%)	6 (86%)
Oxaliplatin-based	4 (15%)	2 (29%)	1 (8%)	1 (14%)
None	3 (11%)	1 (14%)	2 (15%)	0
*Line of treatment*				
1st	5 (19%)	2 (29%)	1 (8%)	2 (29%)
2nd	9 (33%)	2 (29%)	6 (46%)	1 (14%)
≥3rd	13 (48%)	3 (42%)	6 (46%)	4 (57%)
*Best response*				
Stable disease >16 weeks	12 (44%)	1 (14%)	8 (62%)	3 (43%)
Response	15 (56%)	6 (86%)	5 (38%)	4 (57%)
*Progression-free survival*				
Median (weeks, 95% CI)	39.1 (33–46)	45 (42–48)	25.6 (24–27)	38.7 (21–56)

All patients had an initial response to treatment, defined as complete response, partial response or stable disease >16 weeks, and then progressed.
