# Supplementary material for: Acquired RAS or EGFR mutations and duration of response to EGFR blockade in colorectal cancer
Source: Nat Commun. 2016 Dec 8;7:13665. doi: 10.1038/ncomms13665 (PMC5155160; doi:10.1038/ncomms13665)
Supplement: Supplementary Information — Supplementary Figures 1-6 and Supplementary Tables 1-5. [file ncomms13665-s1.pdf]

Supplementary Figure 1

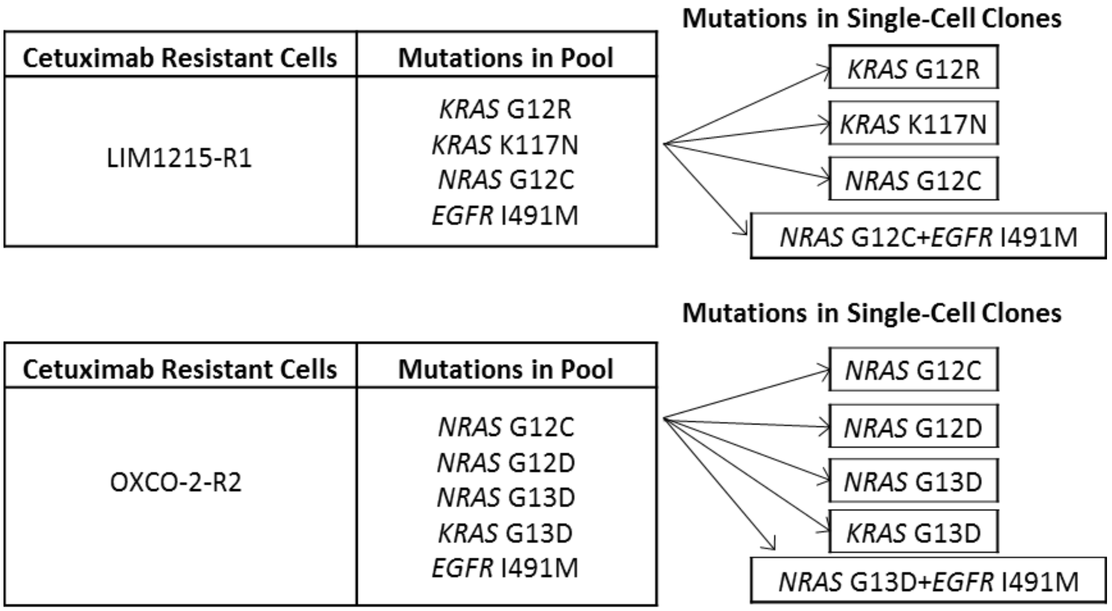

Supplementary Figure 1: Generation of cetuximab-resistant cells and analysis of single-cell clones. Cetuximab-sensitive cells (LIM1215 and OXCO-2) were chronically treated with cetuximab until a resistant population emerged. Single-cell dilution was performed to isolate individual clones. The mutational status of the population and individual clones are indicated.

Supplementary Figure 2

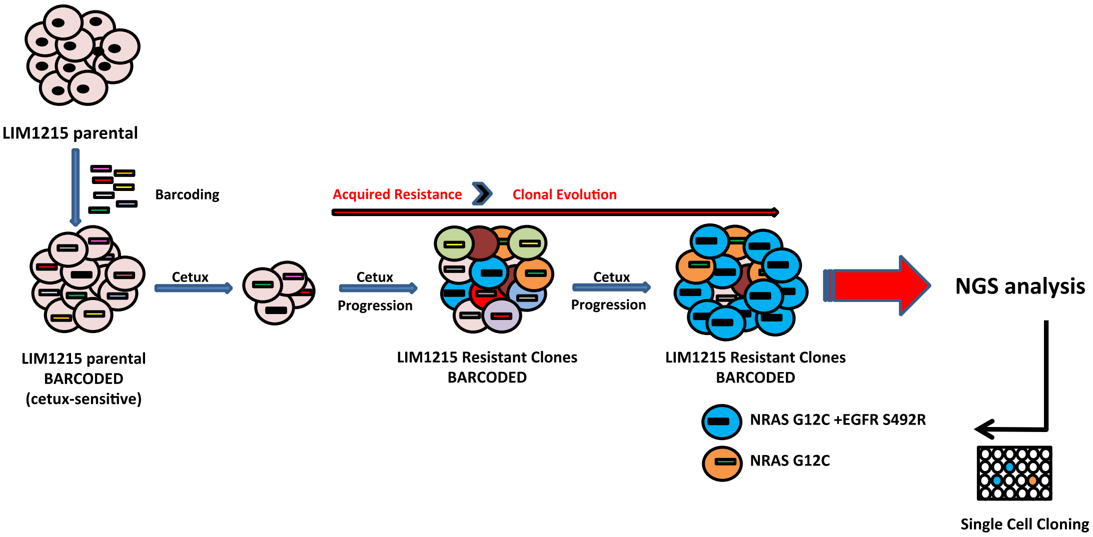

Supplementary Figure 2: Schematic representation of the strategy used to assess clonal evolution during treatment with cetuximab in a population of CRC cells. Cetuximab-sensitive LIM1215 cells were barcoded by means of lentiviral infection and then chronically treated with cetuximab (cetux) until acquired resistance emerged. Resistant cells at different time points were analyzed to monitor clonal evolution during cetuximab treatment. NGS analysis of the last time point (six months) revealed the presence of dominant clones, one of which contained both *NRAS* and *EGFR* mutated alleles as determined by single cell cloning.

Supplementary Figure 3

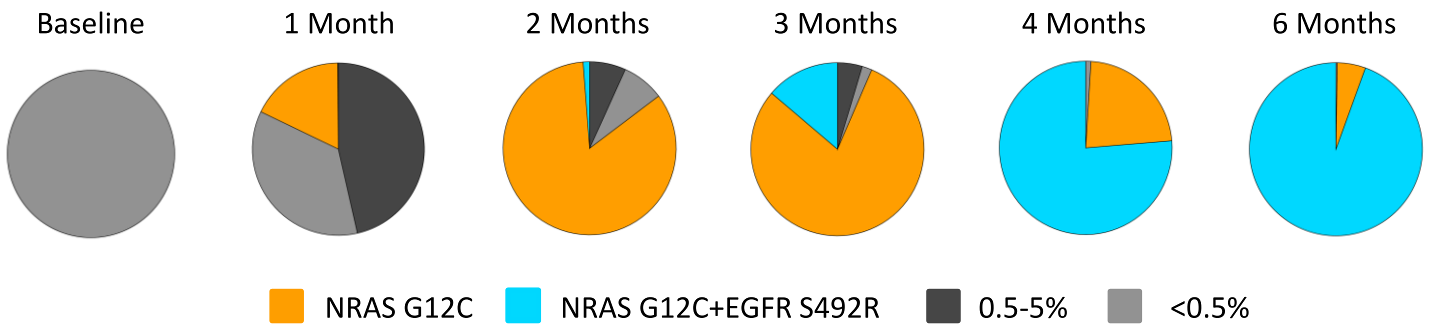

Supplementary Figure 3: Clonal evolution of LIM1215 during EGFR blockade with cetuximab. Barcode and mutational analyses were used to monitor clonal dynamics of LIM1215 CRC cells under cetuximab treatment. Individual colors (orange and light blue) identify unique clones, as determined by barcode profiling, with the indicated mutation identified by sequencing of isolated single cell clones. Light grey indicates barcodes represented at <0.5%. Dark grey indicates barcodes represented at 0.5-5%.

Supplementary Figure 4

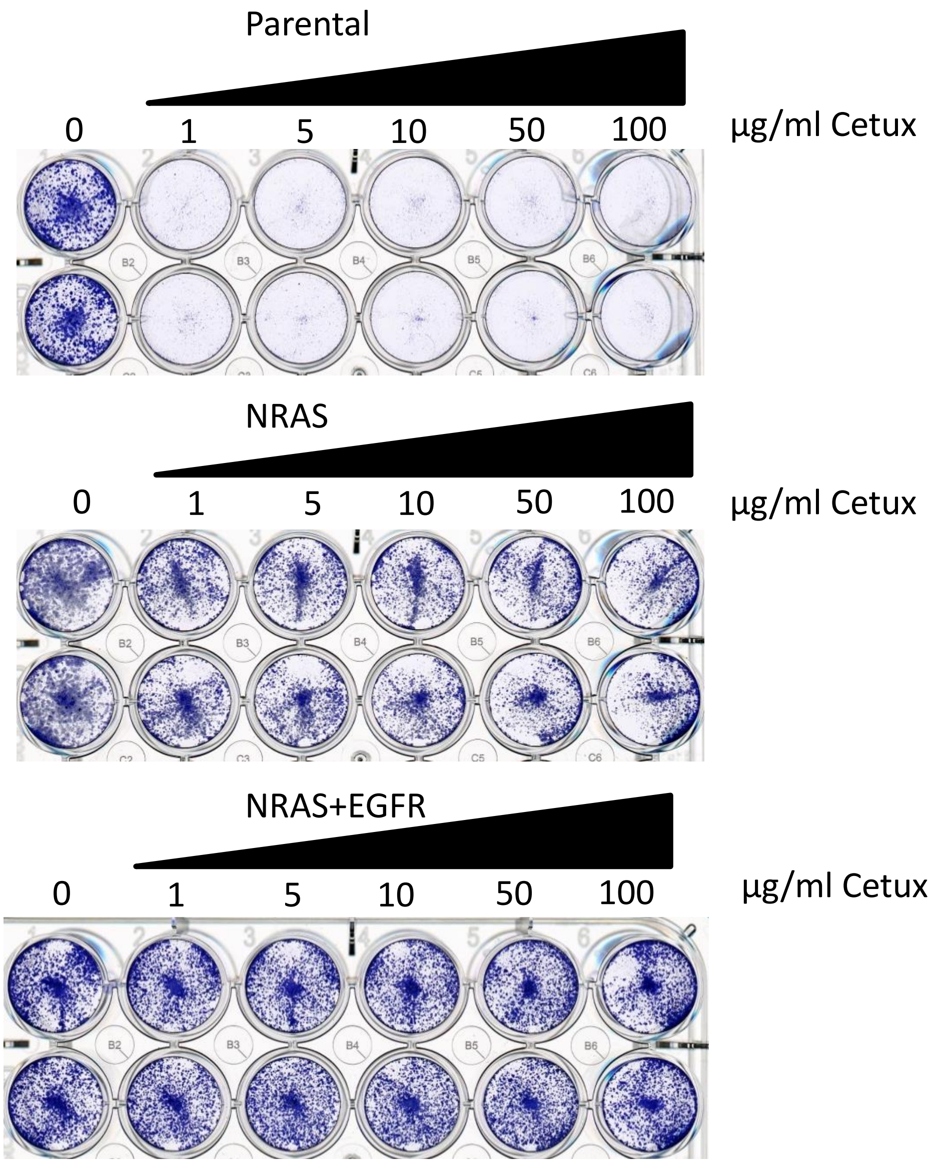

Supplementary Figure 4: Clonogenic assay of single (NRAS G12C) and double mutant (NRAS G12C+EGFR S492R) clones. The indicated cells were treated for eight days with increasing concentrations of cetuximab. At the end of the experiment, cells were fixed and stained with crystal violet solution. Cetux: Cetuximab

Supplementary Figure 5

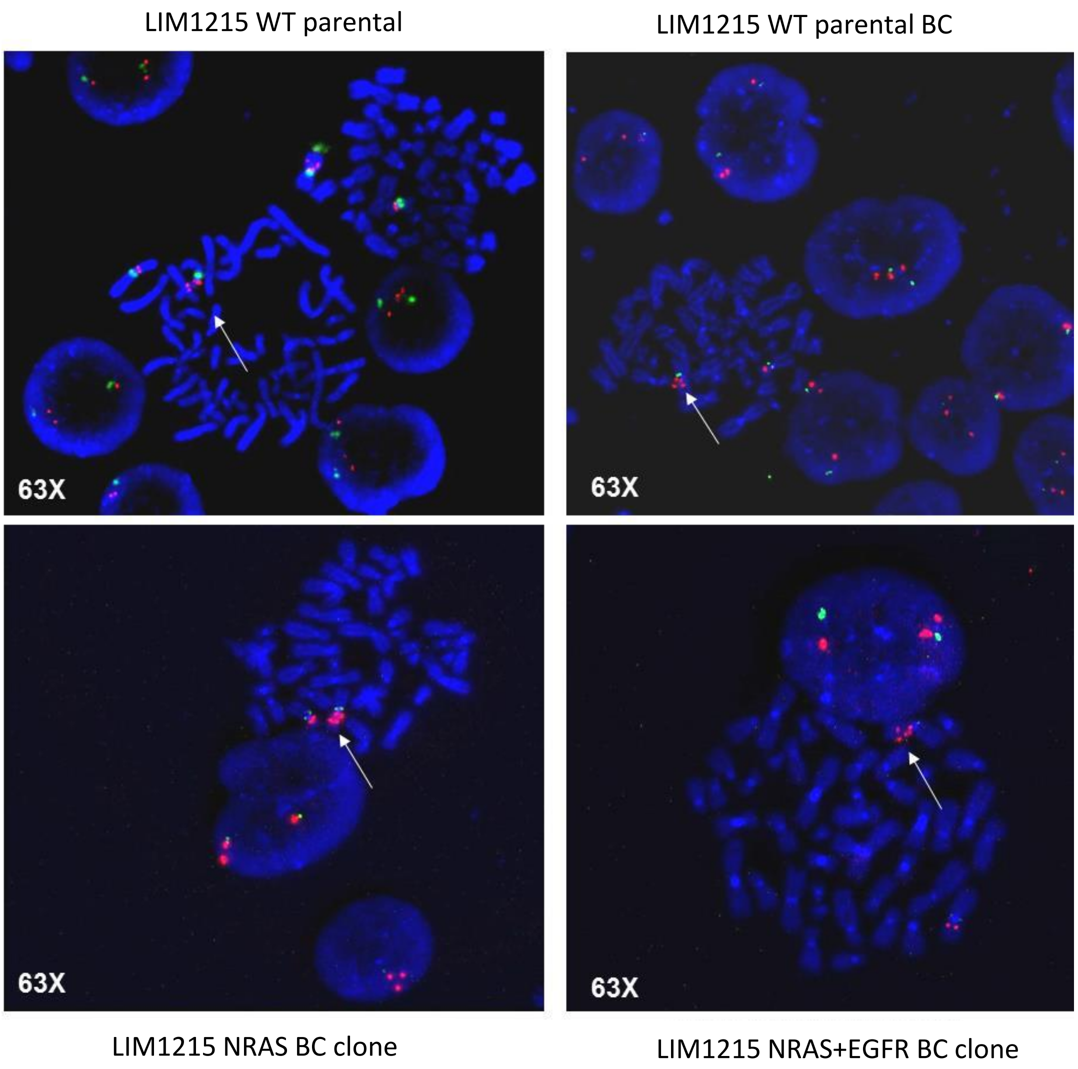

Supplementary Figure 5: FISH measurement of *EGFR* Gene Copy Number. FISH analysis was performed in non-infected parental population of LIM1215, barcoded parental LIM1215, and barcoded resistant LIM1215 clones carrying either *NRAS* G12C or *NRAS* G12C + *EGFR* S492R. Three copies of the *EGFR* gene are present in all cell models. BC: barcoded. Red: *EGFR* gene probe; Green: Chromosome 7 centromeric probe.

Supplementary Figure 6

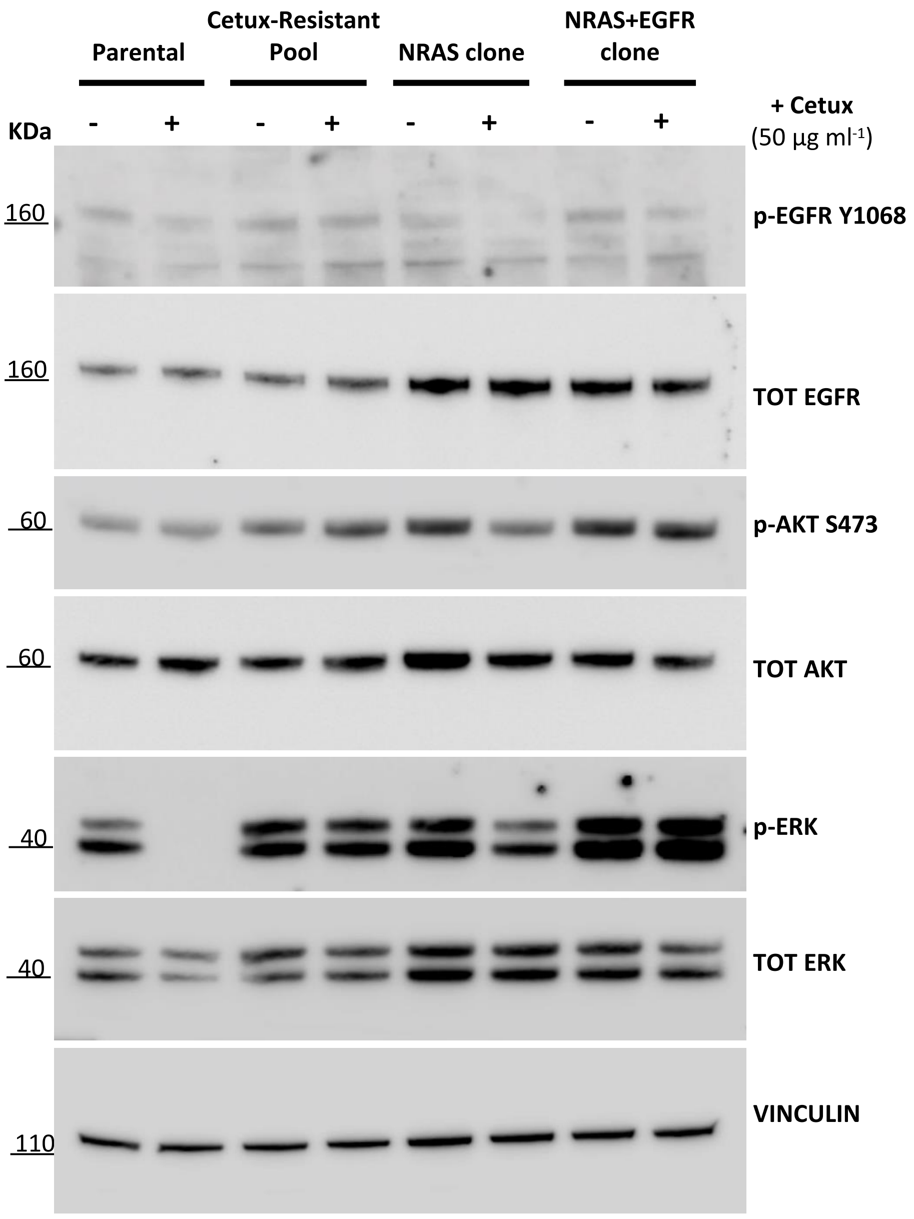

Supplementary Figure 6: Full length of Western blot in Figure 4.

Supplementary Table 1

ID	Baseline Characteristics					*RAS/EGFR* mutations baseline			Metastasic disease				
	Sex	Stage at diagnosis	Primary tumor site	Histology	Primary tumor removed	*RAS* by SoC	*RAS* by NGS	*EGFR* by NGS	Number of metastatic sites	liver	lung	peritoneum	other
1	M	III	unknown	ADC	Yes	wt	wt	wt	1	yes			
2	M	IV	right	ADC	Yes	wt	wt	wt	2	yes		yes	
3	F	IV	rectum	ADC	Yes	wt	*NRAS* G12S	wt	2	yes	yes		
4	F	IV	left	ADC	Yes	wt	wt	wt	4	yes	yes	yes	yes
5	M	III	rectum	ADC	Yes	wt	wt	wt	5	yes	yes		yes
6	M	IV	rectum	ADC	Yes	wt	wt	wt	2	yes	yes		
7	F	III	rectum	ADC	Yes	wt	*KRAS* Q61H	wt	1	yes			
8	F	III	rectum	ADC	Yes	wt	wt	wt	3	yes	yes		yes
9	M	IV	left	ADC	No	wt	wt	wt	4	yes	yes		yes
10	M	IV	left	ADC	No	wt	wt	wt	3	yes		yes	yes
11	F	IV	rectum	ADC	No	wt	wt	wt	2	yes	yes		
12	M	IV	right	ADC	No	wt	wt	wt	1			yes	
13	F	IV	left	ADC	No	wt	wt	wt	1	yes			
14	M	III	rectum	ADC	Yes	wt	*KRAS* Q61L	wt	3	yes	yes		yes
15	M	IV	left	ADC	No	wt	wt	wt	2	yes	yes		
16	M	IV	rectum	ADC	No	wt	wt	wt	1	yes			
17	F	III	rectum	ADC	Yes	wt	*NRAS* Q61K	wt	3	yes			yes
18	F	IV	left	ADC	Yes	wt	NA	NA	2	yes		yes	
19	F	IV	left	ADC	Yes	wt	wt	wt	2	yes		yes	
20	F	IV	left	ADC	Yes	wt	wt	wt	1	yes			
21	M	IV	right	ADC	Yes	wt	NA	NA	2	yes		yes	
22	F	IV	left	ADC	No	wt	NA	NA	2	yes	yes		
23	F	IV	right	ADC	Yes	wt	NA	NA	3	yes	yes		yes
24	M	III	right	ADC	Yes	wt	NA	NA	3	yes	yes		yes
25	M	IV	left	ADC	Yes	wt	NA	NA	1	yes			
26	F	IV	left	ADC	No	wt	NA	NA	2	yes	yes		
27	F	III	rectum	ADC	Yes	wt	NA	NA	3	yes	yes		

Supplementary Table 1: Clinico-pathological characteristics of 27 colorectal cancer patients. M, male; F, female; ADC, adenocarcinoma; SoC, Standard of care; NGS, Next-Generation Sequencing; NA, not available.

Supplementary Table 2

Patient	Institution	Treatment	*KRAS* mutation	*NRAS* mutation	*EGFR* mutation	Response	PFS (w)
1	HMAR	FOLFIRI cetuximab			G465R	SD	63
2	HMAR	FOLFIRI cetuximab	Q61H; V114I		S464L; I491M	SD	39
3	HMAR	IRINO cetuximab		G12S		PR	55
4	HMAR	FOLFIRI cetuximab	G12V			PR	41
5	HMAR	IRINO cetuximab		G60R		SD	24
6	HMAR	IRINO cetuximab	G12V			CR	48
7	HMAR	IRINO cetuximab	Q61H			SD	20
8	HMAR	FOLFOX cetuximab	G12S			SD	24
9	HMAR	IRINO cetuximab	G12A	Q61H		PR	25
10	HMAR	FOLFIRI cetuximab	G12V			SD	23
11	HMAR	IRINO cetuximab	A146T			SD	51
12	HMAR	FOLFOX cetuximab			S492R	PR	42
13	HMAR	FOLFOX cetuximab		Q61L	S492R	PR	60
14	HMAR	FOLFIRI cetuximab	Q61L			SD	26
15	HMAR	FOLFOX cetuximab			S492R	PR	44
16	HMAR	IRINO cetuximab			K467T	PR	52
17	HMAR	IRINO cetuximab	V114I	Q61K		SD	21
18	HMAR	IRINO cetuximab		G12D; G60D	R451C	SD	22
19	HMAR	IRINO cetuximab	G12C; G12D; G12A; G12V		S464L; G465E; G465R	PR	61
20	HMAR	IRINO cetuximab			G465R	PR	45
21	HMAR	IRINO cetuximab			S464L	PR	69
22	ONM	panitumumab			G465R; G465E	PR	39
23	ONM	IRINO panitumumab/ panitumumab IGF1Ri	Q61H	Q61H; Q61L	G465R; S464L	SD	32
24	ONM	panitumumab	Q61H			SD	26
25	ONM	FOLFIRI cetuximab	G12D		G465R; S464L	PR	17
26	SGBH	FOLFIRI cetuximab	Q61H		S464L	PR	67
27	INTM	panitumumab	Q61H			PR	37

Supplementary Table 2: ***KRAS*, *NRAS*, and *EGFR* mutations detected in patients at progression to anti-EGFR therapy.** HMAR: Hospital del Mar (Barcelona, Spain); ONM: Ospedale Niguarda (Milano, Italy), SGBH: Città della Salute e della Scienza, San Giovanni Battista Hospital (Torino, Italy); INTM: Fondazione IRCCS Istituto Nazionale dei Tumori (Milano, Italy); SD: Stable Disease; PR: Partial Response; CR: Complete Response; PFS: Progression-Free Survival; w: weeks.

Supplementary Table 3

Primers for Roche 454 Barcode Sequencing	
Reverse Primer B_Lib-L	CCT ATC CCC TGT GTG CCT TGG CAG TCT CAG ACG AGC ACC GAC AAC AAC GCA GA
Forward Primer A_Lib-L_MID7	CCA TCT CAT CCC TGC GTG TCT CCG ACT CAG **CGTGTCTCTA** TC AAG CAA AAG ACG GCA TAC GAA GAC AG
Forward Primer A_Lib-L_MID13	CCA TCT CAT CCC TGC GTG TCT CCG ACT CAG **CATAGTAGTG** TC AAG CAA AAG ACG GCA TAC GAA GAC AG
Forward Primer A_Lib-L_MID20	CCA TCT CAT CCC TGC GTG TCT CCG ACT CAG **ACGACTACAG** TC AAG CAA AAG ACG GCA TAC GAA GAC AG
Forward Primer A_Lib-L_MID16	CCA TCT CAT CCC TGC GTG TCT CCG ACT CAG **TCACGTACTA** TC AAG CAA AAG ACG GCA TAC GAA GAC AG
Forward Primer A_Lib-L_MID19	CCA TCT CAT CCC TGC GTG TCT CCG ACT CAG **TGTACTACTC** TC AAG CAA AAG ACG GCA TAC GAA GAC AG
**Primers for Sanger Sequencing**	
Gex1-Bpi (Barcode Forward)	TCA AGC AGA AGA CGG CAT ACG AAG ACA
NR2 (Barcode Reverse)	ACG AGC ACC GAC AAC AAC GCA GA
**Primers for Roche 454 GS Junior EGFR ECD Sequencing**	
EGFR1F	GGA GAA ACA AAG TTT TCA GGG ATA CA
EGFR1R	CGG AGG TCC CAA ACA GTT TTT
EGFR2F	GCT CCC TCA AGG AGA TAA GTG ATG
EGFR2R	AAT AAA GGA CCC ATT AGA ACC AAC TC

Supplementary Table 3: **Primer sequences for barcode (Next-Generation and Sanger sequencing) and EGFR analysis.**

Supplementary Table 4

Sample ID	RAS Mutation	Total Copies per Reaction	Fractional Abundance (%)	Sensitivity (%)
LIM1215 Baseline	KRAS G13D	8890	0.15	0.02250
	NRAS G12C	7870	0.06	0.02541
	NRAS G12R	8651	0.07	0.02312

Sample ID	EGFR ECD Mutation	Total Copies per Reaction	Fractional Abundance (%)	Sensitivity (%)
LIM1215 Baseline (first analysis)	R451C	12936	negative	0.01546
	S464L	13068	negative	0.01530
	G465R	14674	negative	0.01363
	G465E	13992	negative	0.01429
	K467T	15158	negative	0.01319
	I491M	13662	negative	0.01464
	S492R	1159	negative	0.17250
LIM1215 Baseline Replicate 1 (second analysis)	R451C	47104	negative	0.00425
	S464L	47938	negative	0.00417
	G465R	47432	negative	0.00422
	G465E	45826	negative	0.00436
	K467T	49984	negative	0.00400
	I491M	45936	negative	0.00435
	S492R	24420	negative	0.00819
LIM1215 Baseline Replicate 2 (second analysis)	R451C	43983	negative	0.00455
	S464L	45364	negative	0.00441
	G465R	44773	negative	0.00447
	G465E	43366	negative	0.00461
	K467T	45716	negative	0.00437
	I491M	43230	negative	0.00463
	S492R	22903	negative	0.00873
LIM1215 Baseline Replicate 3 (second analysis)	R451C	42488	negative	0.00471
	S464L	42724	negative	0.00468
	G465R	43298	negative	0.00462
	G465E	41670	negative	0.00480
	K467T	44594	negative	0.00448
	I491M	41382	negative	0.00483
	S492R	26004	negative	0.00769

Supplementary Table 4: ddPCR for *RAS* and *EGFR* ECD mutations. The second analysis for the *EGFR* ECD was performed using three independently harvested gDNA samples from treatment naïve LIM1215. Each *EGFR* mutation was analyzed in quadruplicate for each baseline. The quantification of the target allele region is presented as the number of Total Copies (mutant plus WT) per sample in each reaction.

Supplementary Table 5

Mutation	ddPCR Probe Sequence or Catalog Number
***KRAS* G12/G13 Multiplex**	ddPCR™ *KRAS* G12/G13 Screening Kit #1863506
***NRAS* G12 Multiplex**	ddPCR™ *NRAS* G12 SCREENING KIT #12001094
***KRAS* Q61 Multiplex**	ddPCR™ *KRAS* Q61 SCREENING KIT #12001626
***NRAS* Q61 Multiplex**	ddPCR™ *NRAS* Q61 SCREENING KIT #12001006
***KRAS* p.G12A**	dHsaMDV2510586
***KRAS* p.G12C**	dHsaMDV2510584
***KRAS* p.G12D**	dHsaMDV2510596
***KRAS* p.G12R**	dHsaMDV2510590
***KRAS* p.G12S**	dHsaMDV2510588
***KRAS* p.G12V**	dHsaMDV2510592
***KRAS* p.G13D**	dHsaMDV2510598
***KRAS* p.Q61K**	dHsaMDS2511862
***KRAS* p.Q61L**	dHsaMDV2010101
***KRAS* p.Q61R**	dHsaMDV2010135
***KRAS* p.Q61H**	dHsaMDV2010131
***KRAS* p.Q61P**	dHsaIS2505058
***KRAS* p.Q61E**	dHsaIS2503954
***KRAS* p.A146T**	dHsaCP2000079
***NRAS* p.G12A**	dHsaMDS42165742
***NRAS* p.G12C**	dHsaMDV2510530
***NRAS* p.G12D**	dHsaMDV2010095
***NRAS* p.G12R**	dHsaMDV2510560
***NRAS* p.G12S**	dHsaMDV2010093
***NRAS* p.G12V**	dHsaMDV2510528
***NRAS* p.G13D**	dHsaCP2500526
***NRAS* p.Q61K**	dHsaMDV2010067
***NRAS* p.Q61L**	dHsaMDV2010069
***NRAS* p.Q61R**	dHsaMDV2010071
***NRAS* p.Q61H**	dHsaMDV2010065
***EGFR* p.I491M**	AATTATGAGCAACAGAGGTG
***EGFR* p.S492R**	TGTTTTCACCTCTGTTTCTTATA
***EGFR* p.G465E**	TTCAGAAAACAAAAATTTGTGC
***EGFR* p.S464L**	AATTTTTGTTTCCTAAAATTATCAC
***EGFR* p.G465R**	TTCAAGAAACAAAAATTTGTGC
***EGFR* p.K467T**	AGCACAAATTTGTGTTTCCT
***EGFR* p.R451C**	TTGAGGGAGCATAATCCC

Supplementary Table 5: ddPCR probe information. For *RAS* multiplex and individual mutations, the catalog number for BioRad is indicated for each probe kit. For *EGFR* mutations, the custom designed probe sequence is indicated
